# Reactivity of Aryl Halides for Reductive Dehalogenation in (Sea)water Using Polymer-Supported Terpyridine Palladium Catalyst

**DOI:** 10.3390/molecules20069906

**Published:** 2015-05-28

**Authors:** Toshimasa Suzuka, Hiromu Sueyoshi, Shohei Maehara, Hiroaki Ogasawara

**Affiliations:** Department of Chemistry, University of the Ryukyus, Okinawa 903-0213, Japan; E-Mails: e123346@eve.u-ryukyu.ac.jp (H.S.); e113331@eve.u-ryukyu.ac.jp (S.M.); rei_sz@yahoo.co.jp (H.O.)

**Keywords:** palladium, polymer-support, hydrodechlorination, seawater

## Abstract

A polymer-supported terpyridine palladium complex was prepared. The complex was found to promote hydrodechlorination of aryl chlorides with potassium formate in seawater. Generally, reductive cleavage of aryl chlorides using transition metal catalysts is more difficult than that of aryl bromides and iodides (reactivity: I > Br > Cl); however, the results obtained did not follow the general trend. Therefore, we investigated the reaction inhibition agents and found a method to remove these inhibitors. The polymeric catalysts showed high catalytic activity and high reusability for transfer reduction in seawater.

## 1. Introduction

Following the adoption of the “Stockholm Convention on Persistent Organic Pollutants” in 2001 many countries prohibited the production and use of chloroarenes, including poly(chlorobiphenyls) (PCB), 3-(3,4-dichlorophenyl)-1,1-dimethylurea and dioxins [1,2]. However, environmental pollution caused by past use of chloroarenes persists. For example, in 2000, Haynes *et al.* reported that 3-(3,4-dichlorophenyl)-1,1-dimethylurea [3], which was widely used in sugarcane (*Saccharum*) plantations, has caused atrophy of coral and seaweed beds in the Australian Great Barrier Reef [4,5,6]. The chloroarenes released into the environment are present at levels of only parts per million in soils and seawater, and the collection and decomposition of chloroarenes is difficult. Therefore, we have attempted to develop a decomposition reaction for chloroarenes in seawater and establish a method to collect the low concentrations of chloroarenes released in seawater [7]. Hydrodechlorination of aryl chlorides has received considerable attention in chemistry, particularly from an environmental perspective. A large amount of research has been devoted to the synthetic application of the reaction as well as to improving its efficiency in homogeneous and heterogeneous conditions [1,2]. In addition, considerable research into the Pd-catalysed dehalogenation reaction in organic solvent and aqueous media [8,9,10,11,12,13,14,15,16,17,18,19,20] has been undertaken since Helquist first reported the reduction of aryl bromides by sodium formate catalysed by Pd(PPh_3_)_4_ in 1978 [21]. 

**Scheme 1:**
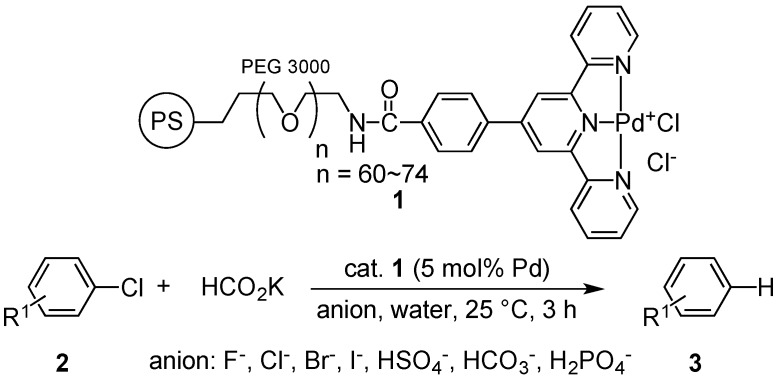
Polystyrene-poly(ethylene glycol)-supported terpyridine-Pd complex and application to the hydrodehalogenation of aryl halides in the presence of anion inhibitors in water.

Our first study into hydrodechlorination of various aryl chlorides in seawater were conducted in the presence of an amphiphilic polystyrene-poly(ethylene glycol) (PS-PEG) resin-bound terpyridine-palladium catalyst and unexpectedly gave dechlorinated products in lower yield than yields in pure water (Table 1) [7]. In these studies, we were surprised to find that the reactive order of halides for the hydrodehalogenation of aryl halides in pure water was Ar–Cl > Ar–Br > Ar–I. For example, the hydrodehalogenation of *p*-bromoacetophenone and *p*-iodoacetophenone for 24 h gave dehalogenated products in 74% and 0.5% yield (Table 3, runs 2–3), although a dechlorinated product was obtained in quantitative yield in the case of *p*-chloroacetophenone [22]. In general, the reductive cleavage of aryl chlorides is more difficult than that of aryl bromides and iodides (reactivity: I > Br > Cl); however, the results obtained did not follow the general trend [8,10,13,23,24,25,26]. Here, we report the anionic influence on the reactivity of hydrodehalogenation in pure water by using a polymer-supported Pd catalyst (Scheme 1). 

## 2. Results and Discussion

We developed the hydrodehalogenation of aryl chlorides, bromides and iodides in water using the polymeric catalyst **1**, which was readily prepared from methyl 4-formylbenzoate, 2-acetylpyridine, NH_4_OH, PS–PEG–NH_2_ resin and (C_6_H_5_CN)_2_PdCl_2_ according to previously reported procedures [27]. The hydrodechlorination of *p*-chloroacetophenone (**2a**) was performed in pure water with HCO_2_K and polymeric catalyst **1** (5 mol % Pd) at 25 °C for 3 h to provide acetophenone (**3a**) in 94% yield. The hydrodechlorination of *p*-chloroacetophenone (**2a**) with HCO_2_K was examined in seawater rather than pure water under similar reaction conditions. The seawater was collected on Kirakira beach in Okinawa. 

Representative results that allow a comparison between the results for seawater and those for water are summarised in Table 1. The reduction of **2a**–**c** with HCO_2_K and polymeric catalyst **1** (5 mol % Pd) in seawater provided acetophenone (**3a**) in 44%–65% yield, whereas, when performed in water, a similar reaction gave 94%–96% yields (Table 1, runs 1–3). The reduction of chlorobenzophenones **2d**–**f** in seawater provided benzophenone (**3b**) in 52%–84% yield (Table 1, runs 4–6, respectively). Reduction of chlorobenzene derivatives **2g**–**l**, which have electron-donating substituents in the *ortho-*, *meta-* and *para-*positions, provided aniline **3c** or phenol **3d** in yields of 9%–90% (Table 1, runs 7–12). Dechlorination of highly chlorinated compounds **2m** and **2n** in seawater gave the corresponding product **3a** in 58% and 90% yield, respectively (Table 1, runs 13–14). It was surprising to find that the reaction efficiency was noticeably affected by whether water or seawater was used. In general, various inorganic salts dissolve in seawater in contrast to pure water. Therefore, we examined halides that dissolve in seawater as catalytic inactivating agents to determine the reactivity of hydrodechlorination in water using a polymer-supported palladium catalyst. The results are summarised in Table 2. The hydrodechlorination of *p*-chloroacetophenone (**2a**) was performed in NaBr solution and NaI solution with HCO_2_K and polymeric catalyst **1** (5 mol % Pd) at 25 °C for 3 h to provide acetophenone (**3a**) in 63% and 66% yields (Table 2, runs 4–5), whereas a similar reaction provided 94%–95% yield in water, NaF solution and NaCl solution (Table 2, runs 1–3).

**Table 1:** Hydrodechlorination of aryl chlorides using polymeric catalyst **1** in (seawater) ^a^. 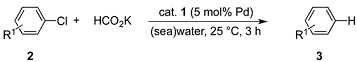

Run	R^1^:2	R^1^:3	Yield (%) (Water)	Yield (%) (Seawater)	Yield (%) (Seawater)
1	*p*-Ac:**2a**	Ac:**3a**	94	56	94 ^c^
2	*m*-Ac:**2b**	**3a**	96	65	95 ^c^
3	*o*-Ac:**2c**	**3a**	95	44	91 ^c^
4 ^b^	*p*-B:**2d**	Bz:**3b**	94	52	87 ^c^
5 ^b^	*m*-Bz:**2e**	**3b**	94	84	84 ^c^
6 ^b^	*o*-Bz:**2f**	**3b**	93	68	68 ^c^
7 ^b^	*p*-NH_2_:**2g**	NH_2_:**3c**	93	9	94 ^c^
8	*m*-NH_2_:**2h**	**3c**	95	44	95 ^c^
9	*o*-NH_2_:**2i**	**3c**	96	33	95 ^c^
10	*p*-OH:**2j**	OH:**3d**	94	90	94 ^c^
11	*m*-OH:**2k**	**3d**	94	77	94 ^c^
12	*o*-OH:**2l**	**3d**	94	59	94 ^c^
13	Ac, 2,5-Cl:**2m**	**3a**	90	21	58 ^c^
14 ^d^	Ac, 2,3,5-Cl:**2n**	**3a**	96 ^e^	43	90 ^e^

^a^ All reactions were performed with **2** (0.2 mmol) and HCO_2_K (0.4 mmol) using polymeric catalyst **1** in H_2_O (1.5 mL) at 25 °C under N_2_. Yields were determined by gas chromatography based on *n*-dodecane as an internal standard; ^b^ 50 µL of toluene was used; ^c^ Reaction time was 12 h; ^d^ 12 mole equivalents (2.4 mmol) of HCO_2_K *vs.* aryl chloride were used; ^e^ Reaction time was 24 h.

**Table 2:** Ionic influence on hydrodechlorination in water using polymeric catalyst **1**. 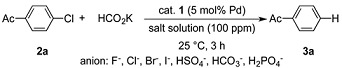

Run	Salts	Amount of Salts (mol) 100 ppm	Yield (%) ^a^
1	none	---	94
2	NaF	3.57 × 10^−6^	94
3	NaCl	2.57 × 10^−6^	95
4	NaBr	1.46 × 10^−6^	63
5	NaI	1.00 × 10^−6^	60
6	Na_2_SO_4_·10H_2_O	0.47 × 10^−6^	96
7	Na_2_CO_3_	1.42 × 10^−6^	95
8	NaH_2_PO_4_·2H_2_O	0.96 × 10^−6^	95
9	AgNO_3_	1.59 × 10^−6^	92
10	AgNO_3_, NaBr	1.50 × 10^−6^	96
11 ^b^	AgNO_3_	1.59 × 10^−6^	0
12 ^b,c^	None	---	94

^a^ All reactions were performed with **2** (0.2 mmol) and HCO_2_K (0.4 mmol) using polymeric catalyst **1** in salt solution of 100 ppm (1.5 mL) at 25 °C under N_2_. The yields were determined by gas chromatography based on *n*-dodecane as an internal standard; ^b^ Seawater was used; ^c^ Reaction time was 12 h.

The reaction proceeded smoothly when a silver bromide ion was precipitated in a silver nitrate solution (Table 2, run 10). These results suggested that Br^−^ and I^−^ were reaction inhibiting agents for the hydrodechlorination of aryl halides in water. Due to the generated Br^−^ or I^−^, the hydrodehalogenation of *p*-bromoacetophenone (**2o**) and *p*-iodoacetophenone (**2p**) did not proceed smoothly, and the desired product was obtained in 74% and 0.5% yield (Table 3, runs 2–3). However, unexpectedly, a quantitative yield of the dechlorinated product was obtained in the case of *p*-chloro-acetophenone (Table 3, run 1). We proceeded to examine the reduction of aryl chlorides by increasing the reaction time under similar conditions. Thus, the reduction of **2a** with HCO_2_K was performed with polymeric precatalyst **1** (5 mol % Pd) in seawater for 12 h to provide acetophenone (**3a**) in 94% yield (Table 2, run 12). All reactions were also carried out in seawater for 12 h to give dechlorinated products in 68%–95% yield (Table 1).

**Table 3:** Hydrodehalogenation of acetophenone-4-halide with ammonium formate in water. 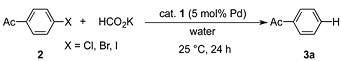

Entry	Aryl Halide	Yield (%) ^a^
1	4-Cl-acetophenone **2a**	quant.
2 ^b^	4-Br-acetophenone **2o**	74
3 ^b^	4-I-acetophenone **2p**	0.5

*^a^* All reactions were performed with **2** (0.2 mmol) and HCO_2_K (0.4 mmol) using polymeric precatalyst **1** in H_2_O (1.5 mL) at 25 °C under N_2_. The yields were determined by gas chromatography based on *n*-dodecane as an internal standard; ^b^ 50 μL of toluene was used.

The recyclability of PS–PEG terpyridine Pd (II) **1** was examined for the hydrodechlorination of *p*-chloroacetophenone (**2a**) in seawater. After the first reaction, which provided acetophenone (**3a**) in 94% yield, the precatalyst was recovered by simple filtration, washed with H_2_O, dried under vacuum, and reused five times under similar reaction conditions to provide **3a** in 95%, 96%, 95%, 95% and 96% yields, respectively. Inductively coupled plasma atomic emission spectroscopy analysis of the aqueous phase revealed barely detectable levels of palladium residue (<1 ppm).

**Scheme 2:**
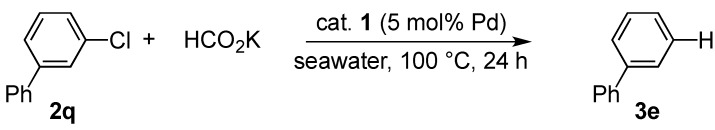
Reduction of PCB.

To investigate the reduction of *meta*-chlorobiphenyl (**2q**) as a representative PCB using polymeric precatalyst **1**, we also tested the reaction with HCO_2_K with polymeric precatalyst **1** in water at 100 °C for 24 h. This reaction provided biphenyl (**3e**) in 91% yield. This result demonstrates the efficient detoxification reaction of PCB in seawater, which is an environmental endocrine disrupter (Scheme 2).

## 3. Experimental Section

### 3.1. General Methods

All manipulations were carried out under aerobic conditions. Water was deionised using a MilliQ gradient A10 apparatus (Millipore, Bedford, MA, USA) to Milli-Q grade. ^1^H- and ^13^C-NMR spectra were recorded in dimethyl sulfoxide-*d*_6_ (DMSO-*d*_6_) at 25 °C on an Avance spectrometer (400 MHz for ^1^H and 100 MHz for ^13^C, Bruker, Karsruhe, Germany). ^13^C-NMR chemical shifts were given relative to the residual DMSO-*d*_6_ peaks used as an internal standard (77.0 ppm and 39.7 ppm, respectively). Mass spectral data were measured on a JMS-T100GCV mass spectrometer (MS) detector and a JMS-T100LP MS detector (JEOL, Tokyo, Japan); the abbreviation “bp” is used to denote the base peak. Gas chromatography (GC) analysis was performed on a GC-2014 GC (Shimadzu, Kyoto, Japan). Infrared spectroscopy (IR) analysis was performed on a FTIR-410 spectrometer (JASCO, Tokyo, Japan).

### 3.2. Materials

The PS-PEG-supported terpyridine ligand was prepared from PS-PEG amino resin (TentaGel *S*-NH_2_, average diameter 90 µm, 1% divinylbenzene cross-linked, loading value of amino residue 0.31 mmol/g, purchased from Rapp Polymere, Tuebingen, Germany) and terpyridine-COOH ligand according to previously reported procedures [28,29].

### 3.3. Synthesis of Polymer-Supported Ligand

A solution of aqueous NH_4_OH (28%, 0.2 mL) and NaOH (80 mg, 2.0 mmol) in minimum water was added to a solution of 4-methoxycarbonylbenzaldehyde (164 mg, 1.0 mmol) and 2-acetylpyridine (242 mg, 2.0 mmol) in EtOH (4.1 mL). After adding NaOH, the solution turned red after 1 h. The solution was stirred vigorously at room temperature in a flask exposed to air for 17 h, after which a yellow suspension was obtained. Water (50 mL) was added, and the solution was neutralised with concentrated HCl to yield a pale yellow precipitate and a red solution. The precipitate was filtered and washed with water. The precipitate was further purified by heating under reflux for 1 h in EtOH (10 mL). The terpyridine-COOH ligand (77.6 mg, 22% yield) was collected by filtration. ^1^H-NMR (DMSO-*d*_6_): δ13.2 (br s, 1H), 8.79–8.76 (m, 4H), 8.69 (d, *J* = 7.9 Hz, 2H), 8.14 (d, *J* = 8.4 Hz, 2H), 8.05 (td, *J* = 7.6, 1.8 Hz, 4H), 7.56–7.53 (m, 2H); ^13^C-NMR (DMSO-*d*_6_): δ166.6, 155.8 (2C), 154.9 (2C), 149.0 (2C), 148.4, 141.0, 136.9 (2C), 132.4, 129.8 (2C), 126.6 (2C), 124.0 (2C), 120.7 (2C), 117.9 (2C); IR (ATR) (cm^−1^): *v* 3414 (br), 3122, 1684, 1565; HR-ESI-MS: calculated for C_22_H_15_N_3_O_2_Na (M + Na) 376.1062, found 376.1061. CAS registry number: 158014-74-5.

### 3.4. Preparation of PS–PEG Resin-Supported Terpyridine Palladium Complex **1**

A Merrifield vessel was charged with PS-PEG-NH_2_ (0.77 g, 0.24 mmol), terpyridine-COOH ligand (127 mg, 0.36 mmol), 1-ethyl-3-(3-dimethylaminopropyl) carbodiimide (138 mg, 0.72 mmol), 1-hydroxybenzotriazole (146 mg, 0.96 mmol) and dimethyl sulfoxide (DMSO) (10 mL). The reaction mixture was shaken at 25 °C for 16 h. The consumption of the primary amino residue of the resin was monitored by the Kaiser negative test. The reaction mixture was filtered, and the resin was washed with DMSO and CH_2_Cl_2_. The resin was dried under reduced pressure to provide the polymer-supported terpyridine (loading value of terpyridine: 0.27 mmol/g, as determined by elemental analysis).

Another Merrifield vessel was charged with resin-supported terpyridine ligand (444 mg, 0.12 mmol) and toluene (10 mL). To this suspension, (C_6_H_5_CN)_2_PdCl_2_ (72.7 mg, 0.19 mmol) was added, and the mixture was shaken (CM-1000) at 25 °C for 2 h. The mixture was filtered, and the resin was washed with toluene and CH_2_Cl_2_. The resulting resin was dried under reduced pressure to provide the polymer-supported palladium complex **1** (loading value of Pd: 0.26 mmol/g). IR (ATR) (cm^−1^); **1**; *v* 2868, 1653, 1453, 1094. *Gel-phase* ^13^C-NMR (DMSO-*d*_6_) δ 164.8, 156.1 (2C), 154.8 (2C), 150.6 (2C), 146.6, 142.1, 137.5 (2C), 136.7, 132.5 (2C), 129.8 (2C), 128.6 (2C), 125.4 (2C), 111.0 (2C), 70.7–69.0 (br, poly(ethylene glycol)).

### 3.5. Hydrodechlorination of Aryl Chlorides

HCO_2_K (67 mg, 0.40 mmol) was added to a mixture of the polymeric precatalyst **1** (40 mg, 0.010 mmol) and *p*-chloroacetophenone (**2a**) (31 mg, 0.20 mmol) in seawater (1.5 mL). The reaction mixture was shaken at 25 °C for 3 h and filtered. The recovered resin beads were rinsed with H_2_O and extracted five times with EtOAc (5 mL). The EtOAc layer was separated, and the aqueous layer was extracted with EtOAc (3 mL). The combined EtOAc extracts were washed with brine (2 mL) and dried over MgSO_4_; then, *n*-dodecane (20 mg) was added. The GC sample was transferred to a GC vial from the organic layer. The yield of acetophenone (**3a**) determined by GC analysis was 94%, with *n*-dodecane as an internal standard. The compounds and CAS registry numbers are as follows: acetophenone (**3a**), benzophenone (**3b**), aniline (**3c**) and phenol (**3d**). CAS registry numbers: 98-86-2, 119-61-9, 62-53-3, and 108-95-2.

## 4. Conclusions

A new polymer-supported terpyridine palladium complex **1** has been developed through the formation of a covalent bound between the resin and the amino group. The resulting resin efficiently catalysed the hydrodechlorination of aryl chlorides with HCO_2_K in seawater under mild conditions to provide the corresponding products in up to 95% yield. We also describe the catalysis inactivation agents and a method to remove these inactivating agents, which were dissolved in seawater for the presented reaction. This is the first example of the hydrodechlorination of aryl chlorides in seawater in which a polymeric precatalyst has been employed. We are continuing to investigate the scope of the hydrodechlorination and possible applications of the precatalyst in other organic transformations.
